# Aged rats have sex-dependent hypothermic effects but not spatial memory impairments following acute alcohol administration

**DOI:** 10.3389/fnbeh.2026.1904796

**Published:** 2026-08-03

**Authors:** Douglas B. Matthews, Megan Schroeder, Kennedy Korger, Olive Schrandt, Michael Tommarello, Katie Johnson, Ann Sobania, Pravesh Sharma

**Affiliations:** 1 Department of Psychology, University of Wisconsin – Eau Claire, Eau Claire, WI, United States; 2 NW Mayo Healthcare, Eau Claire, WI, United States

**Keywords:** aging, alcohol, hippocampus, hypothermia, medial preoptic area, spatial memory

## Abstract

**Introduction:**

The age of the world’s population is continuing to increase, and older individuals are continuing to consume alcohol. Preclinical animal research has demonstrated that aged animals are significantly more sensitive to the effects of alcohol than younger animals and the effect of alcohol in aged animals is often sex dependent.

**Methods:**

The current project investigates if acute alcohol administration produces sex dependent effects in hypothermia and spatial memory impairments in aged female and aged male Sprague Dawley rats.

**Results:**

Acute alcohol administration produced sex dependent effects in that hypothermia in female rats was significantly greater following administration of 1.0 g/kg alcohol but not following 2.0 g/kg alcohol. However, spatial memory impairments were similar between aged female and aged male rats following a 1.0 g/kg alcohol challenge.

**Discussion:**

The current results demonstrate in aged animals that alcohol administration produces sex dependent effects in some tasks and highlight the importance of determining the impact of alcohol in older adults as well as younger subjects.

## Introduction

As the number of older individuals who comprise the world’s and the United States’ populations continues to increase (Statista Research Department, 2026), it is important to understand lifestyle choices that pose health risks to the aging demographic (Tampi et al., 2022). Older individuals use healthcare services significantly more than younger individuals (Lassman et al., 2014; Jones and Dolsten, 2024); therefore, the combined increase in the number of older people and their healthcare needs threatens to overwhelm established healthcare systems (Tampi et al., 2015). Consequently, investigating behaviors detrimental to the aging population is a critical public health need.

Older adults continue to use and misuse alcohol, often at high levels and in dangerous binge drinking patterns (Keyes, 2023; White et al., 2023; MacNeil et al., 2026). While much is known about how alcohol impacts younger individuals (González et al., 2026), there has been less research on the effects of alcohol on older adults (for a review, see Matthews and Imhoff, 2022). Given that alcohol use and misuse pose significant health risks in the aged population due to increased risks of falls and brain bleeds (Zirulnik et al., 2024), increases in cancer (Islami et al., 2024; Esser et al., 2024; Dorobisz et al., 2025) and impaired cognition, and potentially increased dementia (Ho et al., 2022; Anton et al., 2024; Zahr, 2024), it is important to investigate the effects of alcohol in older drinkers (Matthews and Koob, 2023).

To better understand how alcohol affects the aged population, animal models, particularly rodent models such as rats and mice, are often used in research studies. Research using animal models has demonstrated that aged animals are significantly more susceptible to the effects of acute alcohol than younger animals. For example, alcohol induces greater ataxia (Van Skike et al., 2010; Ornelas et al., 2015; Matthews and Mittleman, 2017), increased hypothermia (Watson et al., 2020), more pronounced general learning impairments (Novier et al., 2013; Matthews et al., 2020; Anton et al., 2024), and stronger hypnotic effects (Ornelas et al., 2015; Gano et al., 2017; Perkins et al., 2017) in older animals compared to younger animals. Furthermore, studies also show that aged C57BL/6 J mice self-administer alcohol significantly less than younger mice in the drinking-in-the-dark paradigm; however, their blood alcohol concentrations and total acute alcohol withdrawal over a 10-h test session are similar (Matthews et al., 2026). In addition to increased sensitivity to alcohol in aged subjects, research has also revealed that sex-dependent differences exist in how alcohol impacts aged female and male subjects. Specifically, low doses of alcohol produces a greater anxiolytic effect in aged female rats, while aged male rats exhibit greater motor activity in response to the same doses (Matthews et al., 2024). Finally, aged female rats have significantly less ataxia following an acute alcohol challenge compared to aged male rats (Kerr et al., 2025). It is therefore critical to explore sex differences in aged animals following acute alcohol administration (Matthews and Kerr, 2024; Matthews et al., 2025).

Acute alcohol administration not only produces hypothermia in animals (Abel and York, 1979; Ristuccia and Spear, 2004; Watson et al., 2020) but also impairs spatial memory in rats and mice (Hoffmann and Matthews, 2001; Berry and Matthews, 2004; Van Skike et al., 2019). Furthermore, when spatial memory is tested in the Morris water maze, hypothermia impairs spatial cognition in adult and aged rats (Panakhova et al., 1984; Rauch et al., 1989; Lindner and Gribkoff, 1991). However, the relationship between acute alcohol administration and its sex-dependent effects on hypothermia and spatial memory impairments in aged subjects has not yet been investigated. Consequently, the current project is designed to determine whether acute alcohol causes differential hypothermia and spatial memory impairments in aged female rats compared to aged male rats.

## Methods

### Subjects

The current project used a total of 43 aged Sprague–Dawley rats (consisting of 19 female and 24 male rats) that were obtained from Envigo (Indianapolis, Indiana, United States). They were approximately 12 weeks old at the time of acquisition and were aged to 22 months in the animal colony at the University of Wisconsin – Eau Claire. The mean body weights (± standard error of the mean) at the start of the experimental procedures were 291.2 (9.4) grams for female rats and 577.1 (9.8) grams for male rats. All procedures were approved by the UW – Eau Claire Institutional Animal Care and Use Committee (IACUC). During aging, all subjects had *ad libitum* access to food (Teklad #2018; 18% protein) and tap water, and they underwent daily inspections and weekly cage changes. Subjects were pair-housed throughout aging and the experimental procedure, and they were housed on a 12:12 light–dark cycle (lights on at 6 a.m.). Hypothermic data were collected in the animal colony room under ambient room temperature (~22 ± 2°C).

### Hypothermia

We determined alcohol-induced hypothermia as previously described with minor modifications (Watson et al., 2020). Briefly, on the test day, core body temperature was determined via a digital Physiotemp monitor (Physiotemp Instruments; 154 Huron Avenue, Clifton, New Jersey, 07013, United States) immediately prior to alcohol administration. For each temperature reading, animals were gently hand-held, and an anal probe was inserted into the rectum ~1.25 cm and held until a stable temperature reading was obtained, typically within approximately 5 s. Following baseline core body temperature readings, subjects were randomly assigned (by cage) to receive either a 1.0 g/kg (female *n* = 9 and male *n* = 10) or 2.0 g/kg (female and male *n* = 12) 20% (v/v) alcohol injection intraperitoneally. Following alcohol administration, subjects were placed in their home cages and core body temperature was redetermined every 60 min for the next 180 min.

### Spatial memory test

Three weeks following the hypothermic test, a subset of the subjects underwent a spatial memory test to investigate whether low-dose acute alcohol exposure produces sex-dependent spatial memory deficits in aged animals. Specifically, animals whose hypothermic effect was investigated following the 2.0 g/kg alcohol injection were used to investigate the effect of 1.0 g/kg alcohol on spatial memory. Due to construction disruptions in the building housing the animal colony, we were unable to include all animals tested in the hypothermic study. Animals were trained on the standard spatial memory task in the Morris water maze (Morris et al., 1982). The water maze was 6 ft. in diameter and located in an experimental room rich in extra-maze cues (wall hangings, computer rack, door offset light source). The water of the maze was made opaque by the addition of non-toxic black tempera watercolor paint. The escape platform was made of clear Plexiglas, ~3 in. in diameter, and was located ~1 in. below the water’s surface. Subjects were given four trials per day from the compass points in a random order that was repeated every 4 training days. Subjects were released facing the wall and had 45 s to find and climb onto the platform, where they remained for ~5 s. If the subject did not find the platform, they were gently placed on it for ~5 s. If the subject left the platform, which happened early in training during the 5-s wait period, the subject was replaced on the platform. The performance of subjects was determined by recording the daily average latency to the platform, swim path to the platform, and the swim speed of each subject by an ANY-maze system (version 5.3, Stoelting Co., Wood Dale, IL 60191) via a video camera located above the water maze that was interfaced with a computer located in the room. Subjects were trained for 14 days. Following training, subjects who had an average escape latency of less than 30 s had their spatial memory tested the next day (day 15), 30 min after a 1.0 g/kg intraperitoneal alcohol injection. We chose to test spatial memory 30 min after the 1.0 g/kg intraperitoneal injection to be consistent with our previous studies investigating the effect of alcohol on spatial memory (Matthews et al., 1995, 1999, 2020; Hoffmann and Matthews, 2001; Berry and Matthews, 2004; Chin et al., 2011; Novier et al., 2013).

### Blood alcohol concentration

To determine whether blood alcohol concentrations were different between aged females and aged males during the time point when hypothermia and cognitive data were collected in the current project, immediately following completion of the water maze, subjects had the tip of their tail nicked and a blood sample collected in heparin-coated capillary tubes. Blood samples were centrifuged to separate plasma, and 2.5 μL of plasma was used to determine blood alcohol concentrations using an AM-1 Analox machine (Analox Instruments, Suite 3, Faraday House, King William Street, Amblecote Stourbridge, DY8 4HD) according to the manufacturer’s specifications.

### Statistical strategy

We used two-way analysis of variance (ANOVA) with repeated measures to determine hypothermic differences between males and females across time following alcohol administration. If a significant effect was found, the interaction was further investigated with appropriate *post hoc* tests. For spatial learning and spatial testing following alcohol administration, we used two-way ANOVA with repeated measures and appropriate post hoc tests. Finally, the effect of sex on baseline core body temperature, the differential hypothermic effects of 1.0 g/kg and 2.0 g/kg alcohol, and the effect of sex on blood alcohol concentrations were investigated using independent *t*-tests. All analyses were conducted with GraphPad Prism version 8.4.3.

## Results

### Hypothermia

**Figure 1:**
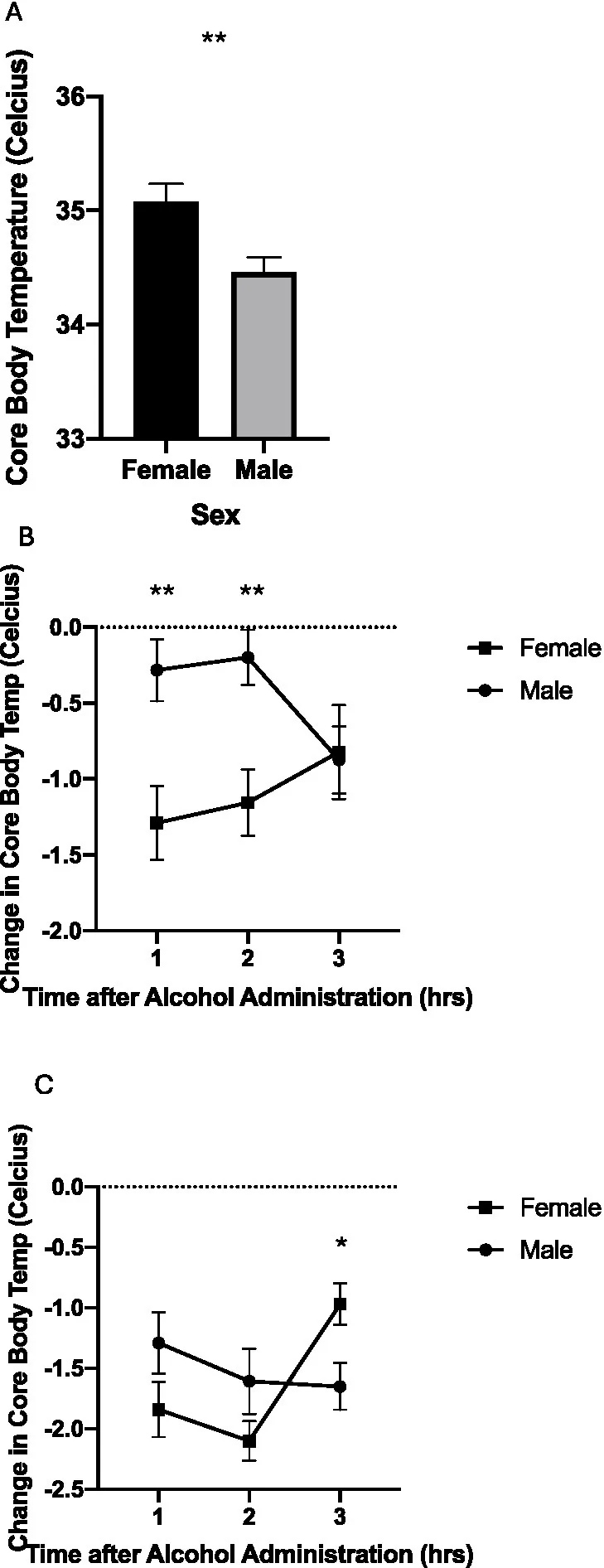
Sex-dependent effects on core body temperature following alcohol administration. **(A)** Baseline core body temperature was significantly higher in aged female rats compared to aged male rats. Scores are the mean core body temperature, and error bars represent the standard error of the mean, **p < 0.01. **(B)** Following administration of 1.0 g/kg alcohol, aged females had significantly greater hypothermia compared to aged males when core body temperature was determined 60 and 120 min later. Data are the change in core body temperature (a negative number indicates a reduction in core body temperature). Values are the mean core body temperature, and error bars indicate the standard error of the mean, **p < 0.01. **(C)** Following administration of 2.0 g/kg alcohol, aged males had significantly greater hypothermia compared to aged females when core body temperature was determined 180 min later. Data are the change in core body temperature (a negative number indicates a reduction in core body temperature). Values are the mean core body temperature, and error bars indicate the standard error of the mean, *p < 0.05.

We first analyzed whether core body temperatures were different based on the sex of the aged subjects and found that aged female rats had significantly higher baseline (pre-alcohol administration) core body temperatures compared to aged male rats (*t*-test, *t* = 2.974, df (41), *p* = 0.0049) (Figure 1A). Because of the significant baseline core body temperature difference between aged females and males (Figure 1A), all subsequent hypothermia analyses were done on changes in core body temperature following alcohol administration (i.e., a difference score) determined by subtracting the pre-alcohol core body temperature from the post-alcohol core body temperature for each of the 1-h data collection points. A negative value indicates hypothermia. We next analyzed whether a dose-dependent effect of alcohol (i.e., 1.0 g/kg vs. 2.0 g/kg) on hypothermia was found 1 h following the alcohol dose regardless of sex, and not surprisingly, found that 2.0 g/kg alcohol produced greater hypothermia than 1.0 g/kg alcohol (*t*-test, *t* = 3.18, df (41), *p* = 0.0028). Due to this significant dose effect and our interest in determining whether sex-dependent effects exist, we analyzed hypothermia following 1.0 g/kg administration separately from hypothermia following 2.0 g/kg administration.

Acute administration of 1.0 g/kg alcohol produced a significant interaction of sex and time following administration in aged female and male rats (two-way ANOVA with repeated measures, sex [2] X time [3], *F* = 6.643, df [2, 38], *p* = 0.0034; main effect of sex, *F* = 5.804, df [1, 19], *p* = 0.0002). Sidak’s multiple comparisons test revealed that the significant difference in core body temperature was driven by lower core body temperatures in aged females at the 1-h time point (*t* = 3.171, df [17], *p* = 0.0165) and the 2-h time point (*t* = 3.369, df [17], *p* = 0.0108) (Figure 1B). In addition, a significant interaction of sex and time was also found following administration of the 2.0 g/kg alcohol injection (two-way ANOVA with repeated measures, sex [2] X time [3], *F* = 15.23, df [2, 40], *p* < 0.0001; main effect of time, *F* = 9.39, df [1, 35], *p* = 0.0008). However, unlike the effects reported with 1.0 g/kg alcohol, hypothermic responses following the 2.0 g/kg alcohol injection were only significantly different at the 3-h time point, when aged males had significantly greater hypothermic responses compared to aged females (Sidak’s multiple comparisons test, *t* = 2.639, df [20], *p* = 0.0465) (Figure 1C).

### Spatial memory

Subjects who were previously tested following a 2.0 g/kg alcohol injection were used to test the effect of the lower dose of alcohol, 1.0 g/kg, on spatial memory in the Morris water maze. Given the importance of testing “cognitively spared” animals, that is, animals that demonstrate learning during the training portion of the task (Matthews et al., 2020; Foster, 2023), we did not test subjects if their average latency score to the submerged platform on the last day of training, day 14, was not less than an average of 30 s. This threshold requirement resulted in seven animals (three females and four males) not being tested in the current project.

**Figure 2:**
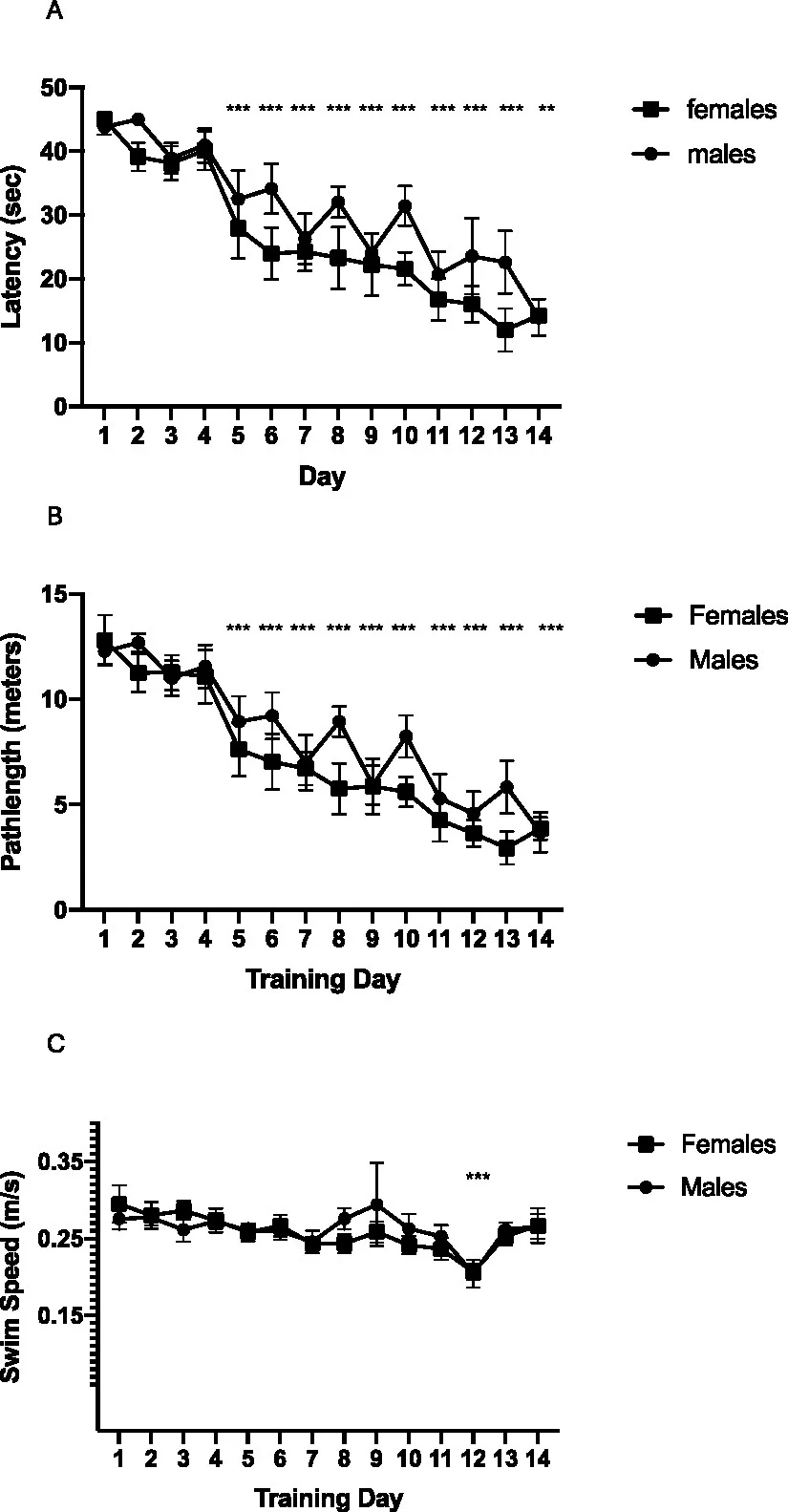
Spatial learning in the Morris water maze in aged female and aged male rats. **(A)** Significant learning of submerged platform location as a function of day. Data are mean latency to the platform per day, in seconds, and error bars are the standard error of the mean. ***p < 0.001 compared to day 1 performance. **(B)** Significant learning of the submerged platform location as a function of day. Data are mean path length in meters to the platform per day, and error bars are the standard error of the mean. ***p < 0.001 compared to day 1 performance. **(C)** Swim speed during training on the submerged platform. Data are mean swim speed to the platform (meters per second per day), and error bars are the standard error of the mean. ***p < 0.001 compared to day 1 performance.

Subjects’ performance significantly improved over the course of the 14-day training procedure as measured by latency to the platform (two-way ANOVA with repeated measures, day [14] by sex [2], main effect of training day, *F* = 19.71, df (13,143), *p* < 0.0001; Dunnett’s multiple comparisons tests compared to performance on training day 1 revealed that all training days from training day 5 to training day 14 were significantly better, training day [TD] 5 q = 4.563, df [143], *p* = 0.001; TD6 q = 4.944, df [143], *p* < 0.001; TD7 q = 6.172, df [143], *p* < 0.001; TD8, q = 5.393, df [143], *p* < 0.001; TD9, q = 6.845, df [143], *p* < 0.001; TD10, q = 5.769, df [143], *p* < 0.001; TD11, q = 8.276, df [143], *p* < 0.001; TD12, q = 7.929, df [143], *p* < 0.001; TD13, q = 8.731, df [143], *p* < 0.001; TD14, q = 9.769, df [143], *p* < 0.001) (Figure 2A). In addition, significant learning was confirmed by pathlength to the platform (two-way ANOVA with repeated measures, day[14] by sex[2], main effect of training day, *F* = 24.09, df (13, 143), *p* < 0.0001; Dunnett’s multiple comparisons tests compared to performance on training day 1 revealed that all training days from training day 5 to training day 14 were significantly better, TD5, q = 4.879, df [143], *p* < 0.001; TD6, q = 5.058, df [143], *p* < 0.001; TD7, q = 6.506, df [143], *p* < 0.001; TD8, q = 5.948, df [143], *p* < 0.001; TD9, q = 7.606, df [143], *p* < 0.001; TD10, q = 6.434, df [143], *p* < 0.001; TD11, q = 8.881, df [143], *p* < 0.001; TD12, q = 9.653, df [143], *p* < 0.001; TD13, q = 9.332, df [143], *p* < 0.001; TD14, q = 10.03, df [143], *p* < 0.001) (Figure 2B). Finally, a significant effect of swim speed during training was found as a function of training day (two-way ANOVA with repeated measures, day [14] by sex [2], main effect of training day, *F* = 3.481, df (13, 143), *p* < 0.0001; Dunnett’s multiple comparisons tests compared to performance on training day 1 revealed that the significant swim speed effect was only found on training day 12, *p* < 0.0001) (Figure 2C).

**Figure 3:**
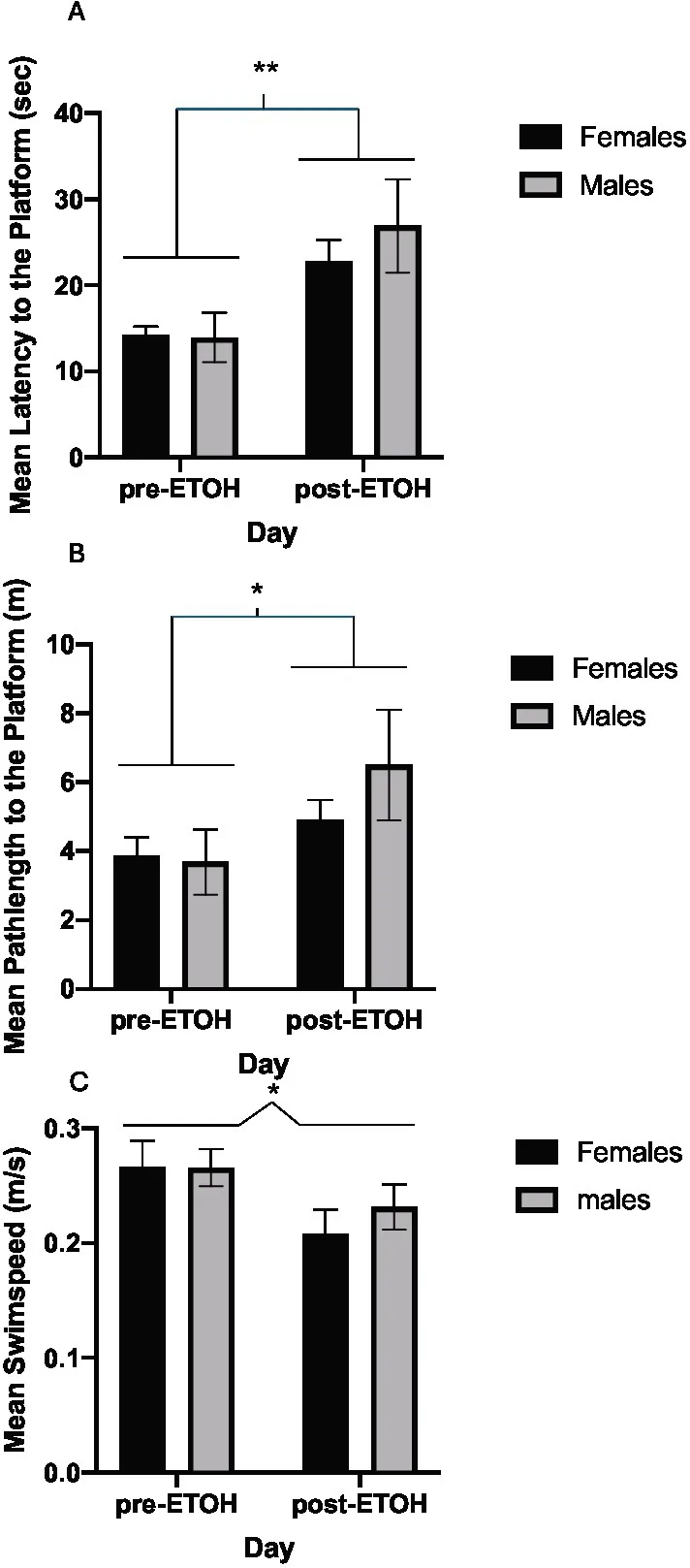
Acute administration of 1.0 g/kg alcohol produces similar spatial memory impairments in aged female and aged male rats. **(A)** Alcohol significantly increased the latency of aged female and male subjects to the submerged platform. Data are mean latency to the platform in seconds, and error bars are the standard error of the mean, **p < 0.01. **(B)** Alcohol significantly increased the swim path length of aged female and male subjects to the submerged platform. Data are the mean path length in meters to the platform, and error bars are the standard error of the mean, *p < 0.05. **(C)** Alcohol significantly decreased the swim speed in meters per second of aged female and male subjects to the submerged platform. Values are mean performance in the water maze, and error bars are the standard error of the mean, *p < 0.05.

To investigate whether 1.0 g/kg alcohol significantly impacted performance on the spatial version of the Morris water maze, we analyzed the performance of subjects 30 min following alcohol administration compared to their performance on the last day of training. As determined by both latency to the submerged platform and swim path length to the submerged platform, 1.0 g/kg alcohol impaired spatial memory performance in both aged female and aged male subjects. Specifically, acute administration of alcohol impaired latency to the platform (two-way ANOVA with repeated measures, day [2] by sex [2], main effect of day, *F* = 9.843, df (1, 11), *p* = 0.0095). In agreement, alcohol administration also increased swim path length to the submerged platform (two-way ANOVA with repeated measures, day [2] by sex [2], main effect of day, *F* = 5.865, *p* = 0.0339). Finally, acute alcohol decreased swim speed (two-way ANOVA with repeated measures, day [2] by sex [2], *F* = 15.2, df (1, 11), *p* = 0.0025) (Figures 3A–C).

### Blood alcohol concentrations

**Figure 4:**
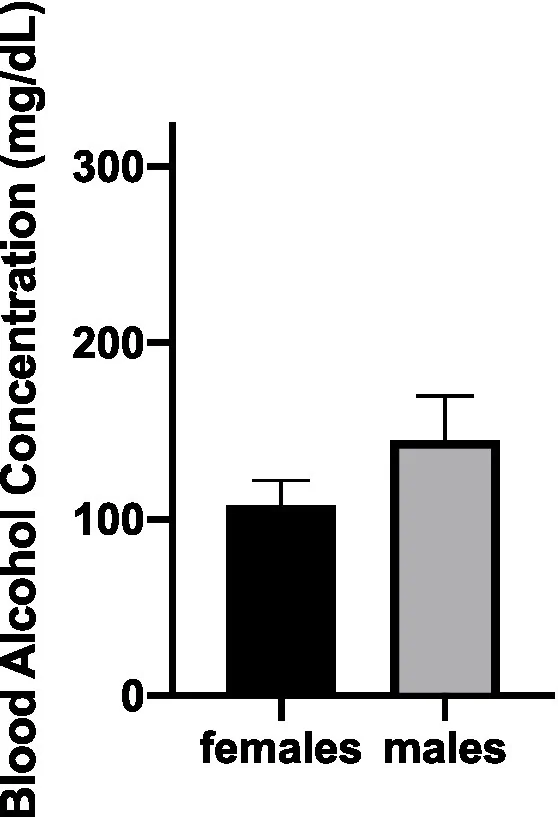
Blood alcohol concentration in aged female and aged male rats following 1.0 g/kg alcohol injection. Values are mean blood alcohol concentration in mg/dL, and error bars are the standard error of the mean.

Supporting previous research, no significant difference was found in blood alcohol concentration between aged females and aged males ~30 min following a 1.0 g/kg alcohol injection (*t*-test, *t* = 1.241, df(11), *p* = 0.2404) (Figure 4).

## Discussion

The present study furthers the understanding of how acute alcohol impacts aged subjects by demonstrating sex- and task-dependent effects in aged female and aged male rats. In the current project, acute alcohol produced hypothermic responses that were greater in aged female rats compared to aged male rats following the administration of 1.0 g/kg alcohol, while producing mostly similar hypothermic effects following the 2.0 g/kg alcohol injection. However, unlike what was found with hypothermia, no significant sex-dependent effect on spatial memory was found following the 1.0 g/kg alcohol challenge. Specifically, alcohol impaired spatial memory to the same levels in aged female and aged male rats in the cognitive test. In addition, the differential task effects and sex-dependent hypothermic effects are not likely due to blood alcohol concentrations, suggesting that the aged central nervous system is different between aged females and males.

The medial preoptic area of the hypothalamus is critical for temperature regulation in mammals (Boulant, 2000), and, as in the current project (Figure 1A), it has been reported that female aged C57BL/6 mice have higher core body temperatures compared to aged male C57BL/6 mice (Sanchez-Alavez et al., 2011). The hypothalamus undergoes significant changes with aging (for a review, see Hajdarovic et al., 2022), which can alter core body temperature in response to ambient temperatures or infections (Vitiello et al., 1986; Norman, 2000). Given the significant difference in hypothermia caused by the 1.0 g/kg alcohol injection, it is likely that aging produces differential effects in the medial preoptic area that are sex-dependent, resulting in differential effects of alcohol on core body temperatures. Further research in this area is needed to identify the specific mechanistic changes underlying age-related alterations in the medial preoptic area.

Unlike the sex-dependent effects of 1.0 g/kg alcohol administration on hypothermia, the same dose of alcohol did not produce sex-dependent effects on spatial memory in the Morris water maze. Specifically, as shown in Figures 3A,B, administration of 1.0 g/kg alcohol impaired spatial memory in aged female and aged male rats as measured by both latency to the submerged platform and swim path length to the submerged platform, but alcohol did not produce a differential effect in aged females compared to aged males. The hippocampus is critical for spatial memory (Morris et al., 1982; Packard et al., 1989; Matthews and Best, 1995), and acute alcohol administration will impair spatial memory performance in the Morris water maze (Berry and Matthews, 2004; Chin et al., 2011). The similar impairment produced in both aged female and male subjects suggests that aging does not differentially alter the effect of alcohol on hippocampal-dependent cognitive function, at least when lower doses of alcohol are used. It is interesting that a significant impairment in spatial memory was found following the 1.0 g/kg challenge in aged animals, though, considering spatial memory impairments in adult animals are not found unless the dose of alcohol is greater than 1.25 g/kg alcohol (for a review, see Van Skike et al., 2019). This suggests that aging alters hippocampal function in such a way as to increase sensitivity to acute alcohol. In addition, it has previously been found that acute alcohol degrades the spatial specificity of hippocampal place cells recorded in awake, freely behaving rats (Matthews et al., 1996; White and Best, 2000), and based on the current results, it is predicted that lower doses of acute alcohol will degrade the spatial specificity of hippocampal place cells significantly more in aged animals compared to young animals.

It is possible that, in aged female and male rats, a movement impairment (i.e., ataxic effect) due to the 1.0 g/kg alcohol injection confounded the reported spatial memory impairment in the water maze. For example, we previously demonstrated that 1.0 g/kg alcohol will produce ataxic impairments in aged Sprague–Dawley rats that are sex-dependent, with aged males having a significantly larger ataxic effect than aged females (Kerr et al., 2025). However, we do not believe an ataxic effect is confounding the reported spatial memory impairment in the present study. First, Figure 3A shows a spatial memory impairment when latency to the platform is measured due to alcohol injection, and Figure 3C shows that alcohol administration also reduced swim speed. This could show a potential confounding as outlined above. However, Figure 3B also demonstrates a spatial memory impairment when swim path length is used as a measure of spatial memory performance. Swim path length is independent of movement deficits and/or swim speed; thus, the agreement between a spatial memory impairment measured by latency and one measured by path length strongly supports the conclusion that acute alcohol impairs spatial memory. Second, previous research found that aged males are significantly more ataxic compared to aged females when administered 1.0 g/kg alcohol. The current spatial memory results show a similar impairment produced by 1.0 g/kg alcohol in aged males and females, further supporting the conclusion that alcohol impairs spatial memory in aged animals and that this effect is not confounded by a general movement impairment.

Previously, it has been reported that hypothermia impairs spatial cognitive performance in the Morris water maze in adult and aged rats (Panakhova et al., 1984; Rauch et al., 1989; Lindner and Gribkoff, 1991). The current project demonstrates that low-dose alcohol produces hypothermia and impairs spatial memory in the Morris water maze. This suggests that at least part of the current spatial memory impairment may be due to the hypothermic effect produced by acute alcohol. However, the interaction of alcohol, hypothermia, and spatial memory performance is complex due to the following factors. First, previous research demonstrated that administration of 1.0 g/kg alcohol did not significantly impair non-spatial performance in the water maze in aged animals compared to younger animals (Kerr et al., 2025). This suggests that hypothermia produced by alcohol administration does not produce a global cognitive impairment. Second, the current results reveal a sex difference in hypothermia produced by 1.0 g/kg alcohol administration but no sex difference in spatial memory produced by 1.0 g/kg alcohol. The difference in the effect of alcohol on the two measures suggests that the reported spatial memory impairment is not due just to hypothermia and that the potential interaction of alcohol, age, and hypothermia on spatial memory is complex and in need of additional research.

It is unlikely that the differential effect of 1.0 g/kg alcohol between the hypothermic test and the spatial memory test is due to differential uptake or metabolism of alcohol between aged female and aged male subjects. As shown in Figure 4, blood alcohol levels were similar between the aged female and aged male subjects immediately following the spatial memory test. The similar blood alcohol levels agree with previous studies from our laboratory demonstrating similar blood alcohol levels in aged female and aged male Sprague–Dawley rats (Kerr et al., 2025).

The current project has limitations. First, the study was designed to only investigate sex differences in aged animals without including younger animals. This does not allow for a direct comparison of sensitivity to the drug across ages. However, we and others have previously demonstrated that aged animals are significantly more affected by acute alcohol compared to younger animals (Van Skike et al., 2010; Novier et al., 2013; Ornelas et al., 2015; Gano et al., 2017; Perkins et al., 2017; Watson et al., 2020; Kerr et al., 2025; for a review, see Matthews et al., 2025). As such, it is reasonable to believe that aged animals would exhibit greater hypothermic and spatial memory effects than younger animals. Second, in our previous hypothermic study, we measured the effect of acute alcohol exposure for 360 min (Watson et al., 2020), whereas in the current project we measured hypothermia for only 180 min. However, in our previous study (Watson et al., 2020), core body temperatures had mostly returned to baseline after 180 min following 1.0 g/kg and 2.0 g/kg injections; therefore, we only determined temperature values in the current project up to 180 min. Third, the effect of alcohol administration on spatial memory was determined after the effect of alcohol on hypothermia, and carryover effects are possible. Finally, we did not determine the effect of 2.0 g/kg alcohol on spatial memory due to the time constraints caused by construction in the building where the animal facility is located.

We and others have previously demonstrated sex-dependent effects of acute alcohol on aged animals. Specifically, sex-dependent effects following alcohol administration have been found in loss of righting reflex (Gano et al., 2017; Perkins et al., 2017), ataxia (Kerr et al., 2025), and anxiolytic and motoric activity (Matthews et al., 2024). Given the continued alcohol consumption in the aged population, future research is needed, particularly studies that identifies central nervous system mechanisms underlying differential effects of alcohol on aged females compared to aged males.

In conclusion, the current study furthers our understanding of how acute alcohol differentially impacts aged subjects by revealing sex-dependent effects in aged female rats compared to aged male rats on core body temperature. Furthermore, the current data highlight that the sex-dependent effects are task-dependent in that no significant difference was found in a spatial memory task. Given the increase in the number of older individuals in most countries, continued investigations into the effects of drugs, such as alcohol, are warranted.

## Data Availability

The raw data supporting the conclusions of this article will be made available by the authors, without undue reservation.
